# Case Report: sintilimab-induced Stevens-Johnson Syndrome in a patient with advanced lung adenocarcinoma

**DOI:** 10.3389/fonc.2023.912168

**Published:** 2023-09-14

**Authors:** Xueqin Li, Guanghui Li, Diangang Chen, Linxi Su, Ru-peng Wang, Yi Zhou

**Affiliations:** ^1^ Institute of Cancer, Xinqiao Hospital, Army Medical University, Chongqing, China; ^2^ Department of Pathology, Xinqiao Hospital, Army Medical University, Chongqing, China; ^3^ Department of Rheumatology and Dermatology, Xinqiao Hospital, Army Medical University, Chongqing, China

**Keywords:** immunotherapy, adverse events, Stevens-Johnson Syndrome, sintilimab, case report

## Abstract

Immune checkpoint inhibitors (ICIs) have been widely applicated in clinical therapy in recent years. Skin-related adverse reaction is one of the most common adverse events for ICIs. Stevens-Johnson syndrome (SJS) is one of the serious cutaneous reactions threatening the life. Here, we reported a case of 76-year-old male patient with poorly differentiated metastatic lung adenocarcinoma, after 9 weeks exposure of sintilimab (3 doses) combined with paclitaxel liposome after concurrent chemotherapy/radiotherapy, experienced Stevens-Johnson syndrome involving limbs, trunk, lip and the oral mucosa. Biopsy of the skin tissue showed infiltration of CD4 and CD8 positive T lymphocytes. We also found PD-L1 expression in the glands and the basal layer of the skin. This finding is distinct from the previously reported expression of PD-L1 on the surface of epidermal keratinocytes in patients with SJS due to immunotherapy.

## 1 Introduction

Immune checkpoint inhibitors (ICIs) are effective in the treatment of tumors and have been widely explored in recent years. ICIs kills tumors by activating autoimmune cells that may damage tissue. Due to their uniqueness, the side effects of ICIs are different from those of radiotherapy and chemotherapy. Skin toxicity is one of the most common adverse reactions for ICIs. Here, we presented a case of Stevens-Johnson syndrome (SJS) with poorly differentiated lung adenocarcinoma after being treated by sintilimab. Biopsy of the skin tissue showed infiltration of CD4 and CD8 positive T lymphocytes and PD-L1 expression in the glands and the basal layer of the skin.

## 2 Case description

**Figure 1:**
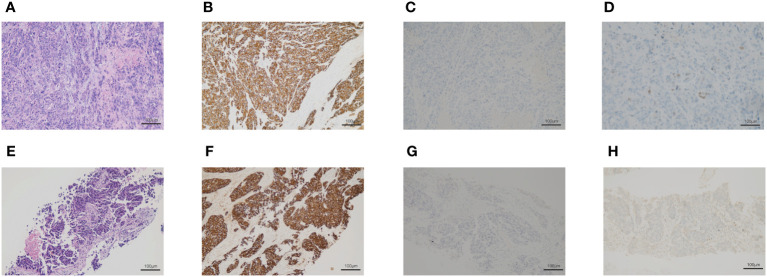
The biopsy of the right supraclavicular lymph node. **(A–D)** The first biopsy of the right supraclavicular lymph node. **(A)** Hematoxylin and eosin-stained staining of the right supraclavicular lymph node. **(B)** Immunohistochemical staining of CK. **(C)** Immunohistochemical staining of p63. **(D)** Immunohistochemical staining of TTF-1. **(E–H)** The second biopsy of the right supraclavicular lymph nodes. **(E)** Hematoxylin and eosin-stained section of the right supraclavicular lymph node. **(F)** Immunohistochemical staining of CK. **(G)** Immunohistochemical staining of p63. **(H)** Immunohistochemical staining of TTF-1.

On March 2020, a 76-year-old male with a long history of heavy smoking (about 75 pack-years) started to develop irritating cough, nausea, and paroxysmal chest tightness. These symptoms gradually worsened in the following months. On June 2020, positron emission tomography/computed tomography (PET/CT) scan was performed, revealing a lesion in the apical segment of the upper lobe of the right lung (5.1cm× 3.3 cm), encircling the superior vena cava and right pulmonary artery, with possible mediastinal, and right upper and lower clavicular lymph node metastases. Tumor marker carcinoembryonic antigen (CEA) was 183.4ng/mL. An electrocardiogram was also conducted, suggesting anterior interstitial and anterior wall old myocardial infarction, but the patient has no symptoms. Then, we performed a biopsy of the right supraclavicular lymph nodes, and histopathologic analysis suggested a poorly differentiated carcinoma, with immunohistochemical staining showing CK (+), CK 7(−), CK20 (−), P63 (−), TTF-1 (−), NAPSINA (−), Ki-67 (60%+) (**Figures 1A–D**). Next-generation sequencing (NGS) analysis was performed and identified. The results were obtained: EGFR, ALK, RET, MET, ROS1 without positive driver genes, the tumor proportion score (TPS) of PD-L1 protein expression was less than one percent, microsatellite state was stable (MSS), and tumor mutation burden (TMB) was 22.19 mutations/Mb. The clinical stage was cT4N3M0. Concurrent chemotherapy/radiotherapy was administered from July 24 to August 13, 2020. The radiotherapy target included the lung mass, right hilar, mediastinal, supraclavicular and subclavian lymph nodes and the dose of PGTV was 45Gy/15F. On July 20 and August 12, 2020, two cycles of paclitaxel liposome and carboplatin were performed. There were no obvious side effects during the treatment. One month after chemoradiotherapy, chest CT scan was performed to evaluate efficacy and the tumor regression degree was 31.4%. Then, the treatment was converted to paclitaxel liposome combined with sintilimab immunotherapy on September 17. After three cycles, the lesion was assessed as stable.

**Figure 2:**
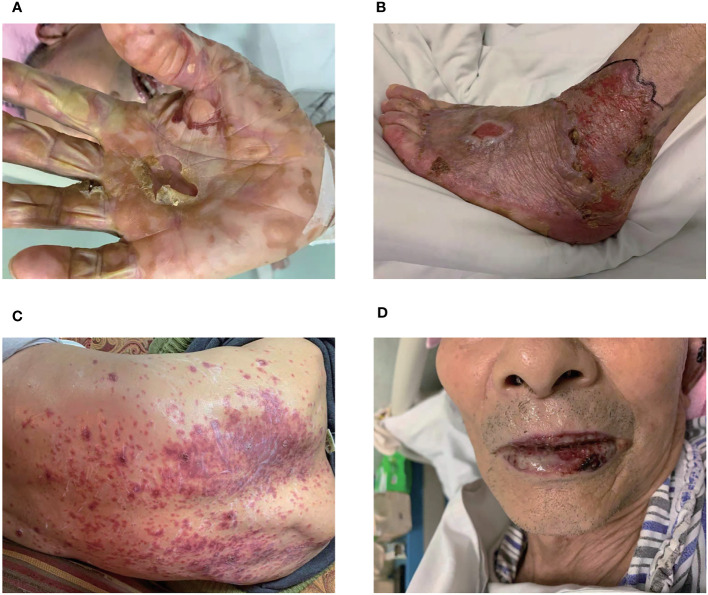
Skin and lips mucosa injury. The localized skin lesions presented with erosions on hands **(A)** and feet **(B)**. **(C)** The erythema was on the back. **(D)** The lips mucosa had painful erosions.

**Figure 3:**
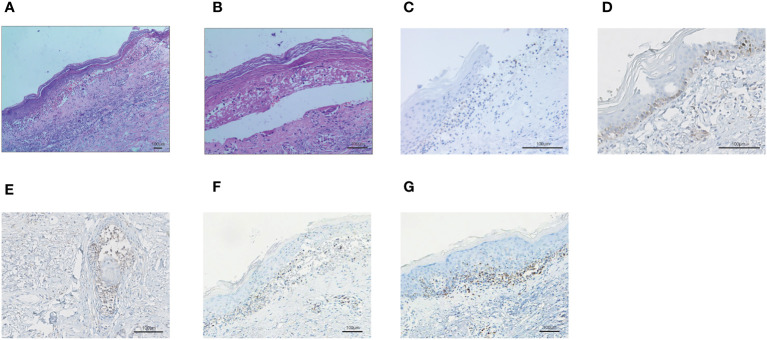
The biopsy of the skin lesion of right forearm. **(A-B)** Hematoxylin and eosin-stained staining of skin biopsy. The biopsy of the skin lesion showed local keratinocytic necrosis **(A)** and subepidermal split **(B)**. **(C)** PD-1 expression in skin lesion. **(D)** PD-L1 expression in the basal layer of the skin. **(E)** PD-L1 expression in the glands of the skin. **(F)** CD4+ T lymphocyte infiltration in the skin. **(G)** CD8+ T lymphocyte infiltration in the skin.

On November 19, 2020, patient began to develop painful erosion of the lips and the oral mucosa, dark red erythema localized to the chest wall, back, hands and feet. Hands and feet started to be swollen and blistered (**Figure 2**). On November 24, 2020, the patient was admitted to our dermatology department. TOX IgM, FZ IgM, CMV IgM, DP IgM were all negative. HCMV-DNA, EBV nucleic acid, BK virus nucleic acid, and JC virus nucleic acid were also all negative. After treated with antihistamines and compound glycyrrhizin anti-inflammatory therapy, the symptoms were not relieved and the pain of the lips and oral mucosa was getting worse. A pathological examination of the right forearm was performed on November 26, 2020. It showed epidermal necrosis and subepidermal split (**Figures 3A, B**), consistent with SJS. Immunofluorescence showed granular IGM and linear deposits on the basement membrane zone. Staphylococcus aureus was detected in the pustules. Severe drug rash with infection was considered and intravenous shock treatment was administered with methylprednisolone 50 mg/day (1 mg/kg/day) and infusion of immunoglobulin (**Supplementary** **Table S1**). Mupirocin ointment was applied to the infected area. 20 days later, the skin erythema became lighter, and the erosion and desquamation appeared on the chest, back and upper extremities. Some of the toenails became yellow and thickened before falling off. The dose of prednisone was reduced to 40 mg/day orally, until discontinuation on January 11, 2021. However, the dorsum of the left foot and the left external ankle were still erosional, the wound discharge was reviewed without bacterial or fungal growth. A medical wound nursing membrane was applied to promote healing, and the erosion had improved but still persisted. The erosion was somewhat resistant to local corticosteroids. Then we analyzed the patient’s right forearm skin biopsy to assess for PD-1 and PD-L1. The results were positive for both PD-1 (**Figure 3C**) and PD-L1 expression (22C3 pharmDx assay, Agilent Technologies), particularly with PD-L1 expression in the basal layer of the skin (**Figure 3D**) and in the glands (**Figure 3E**). PD-L1 expression in tonsil tissue was used as a control to verify antibody specificity (**Supplementary Figure 1**). We also performed CD4 and CD8 positive T lymphocyte expression assay, and the results showed positive CD4 (**Figure 3F**) and CD8 (**Figure 3G**) positive T lymphocyte infiltration.

After two months, chest CT revealed that the right lung lesion and right supraclavicular lymph nodes were significantly larger than before. We performed a second biopsy of the right supraclavicular lymph node and confirmed a poorly differentiated adenocarcinoma with immunohistochemical staining results showing CK 7(−), TTF-1 (+), NAPSINA (−), P40 (−), P63(−), CK5/6(−), 35BH11(+), CK (+), Ki-67 (70%+) (**Figures 1E–H**). Two cycles of chemotherapy with pemetrexed were administered, however the tumors continued to grow and the patient’s physical state was not adequate for further chemotherapy. On July, 2021, chest CT revealed tumor progressed. On August, 2021, the right lung lesion and right supraclavicular lymph node were implanted with radioactive iodine-125 particles. The patient was followed up by telephone in December 2021, and he was in a good condition.

## 3 Timeline

**Figure 4:**
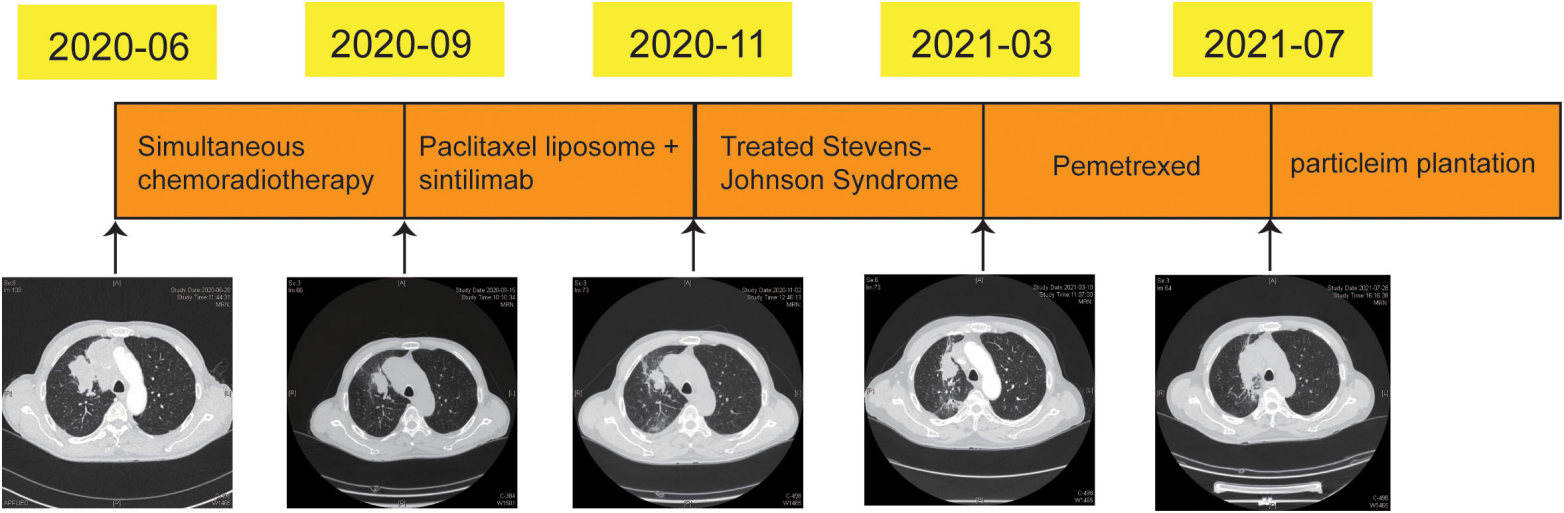
Timeline of treatment and diagnostic assessment of lung lesions during treatment.

On June 2020, the patient was diagnosed with poorly differentiated lung cancer and the clinical stage was cT4N3M0. From July to August 2020, concurrent chemotherapy/radiotherapy was administered. Paclitaxel liposome and sintilimab were administered for three cycles from September 2020. Unfortunately, SJS-induced by sintilimab on November 2020. Then, the treatments for SJS were performed. on March 2021, the right lung lesion and supraclavicular lymph node were significantly larger than before, two cycles of chemotherapy with pemetrexed were administered. On July 2021, the tumor was progression again and the radioactive iodine-125 particles were implanted (**Figure 4**).

## 4 Diagnostic assessment

On June 28, 2020, the mass in right lung approximately 5.1cm × 3.3 cm. On September 15, 2020, the tumor regression degree was 31.4% after chemoradiotherapy (approximately 3.5 cm × 2.2 cm). On November 2, 2020, the tumor assessment was stability (approximately 3.5 cm × 2.2 cm). On March 9, 2021, the tumor mass of right lung (approximately 4.2 cm ×2.8 cm) and right supraclavicular lymph nodes were significantly larger than before. On July 28, 2021, the tumor of right lung was significantly larger than previous detection (approximately 7.2 cm ×3.6 cm) (**Figure 4**).

## 5 Discussion

ICIs, including the PD-1, PD-L1, and CTLA-4 monoclonal antibodies, can result in impressive response rates and durable disease remission in patients with cancer and have thus revolutionized the treatment of advanced cancer patients. There are several biomarkers that can be used to predict the efficacy of immunotherapy, including PD-L1, TMB, MSI, and dMMR. Checkmate-227 study on non-small cell lung cancer (NSCLC) showed that high TMB is a positive predictor for the efficacy of PD-1 antibodies, especially with the use of combination therapy (1). In this case, tumor tissue NGS analysis before treatment suggested high TMB, and we provided treatment with sintilimab, which is a monoclonal antibody against PD-1 and had been performed to treat non-small cell lung cancer (2).

Cutaneous adverse reactions have been the most common and increase with continued use of ICIs. The common skin manifestations are maculopapular rash, pruritus, and vitiligo-like lesions. Other potentially severe cutaneous adverse events include multiforme-like drug reaction, Stevens-Johnson syndrome (SJS), and drug rash with eosinophilia and systemic symptoms (DRESS syndrome) (3–5). SJS was severe adverse cutaneous drug reactions that predominantly involve the skin and mucous membranes. The damage is primarily to the body surfaces, with painful red spots and blisters forming on the skin, eyes, mouth, and genitals. Ma et al. reported ocular side effects of 8 cancer patients in whom SJS/TEN developed during ICIs treatment. The ocular manifestations included no involvement in 3 patients (37.5%), mild involvement in 2 patients (25%), and most severe involvement in 3 patients (37.5%) (6). In our study, the disease mainly affected the chest, back, limbs and lip, but did not involve the patient’s eyes. According to the patient’s clinical stage, we formulated a treatment plan of concurrent chemotherapy/radiotherapy followed by immunotherapy maintenance (7). For PD-L1 <1%, durvalumab has no or insignificant benefit from available data (8), so we adopted the mode of chemotherapy combined with immunotherapy. Due to the efficacy, cost - effective and accessibility of drugs, we chose sintilimab (2). The patient developed painful erosion of the lips and the oral mucosa, severe and extensive skin lesions after 3 cycles of treatment with sintilimab, a pathological examination of right forearm showed epidermal necrosis and subepidermal split, which was consistent with SJS. Generally speaking, IGM deposit was undetected in SJS. Autoimmune-related indexes were tested and the result showed SSA antibody (3+), RO-52 (3+), antinuclear antibody titer (1:100) and ANA-PH-S (coarse particle type). These might lead to granular IGM and linear deposits of Immunofluorescence on the basement membrane zone.

According to the literature, many different classes of drugs have been found to cause SJS/TEN. Drugs that bear a high risk for SJS/TEN including antileptics (carbamazepine, lamotrigine, phenobarbital and phenytoin), anti-infective drugs (sulfonamides, sulfasalazine and nevirapine), NSAIDs (piroxicam) and allopurinol. Other drugs are moderate risk for SJS/TEN, such as antibiotics (cephalosporins, macrolides, Quinolones, Tetracyclines) and NSAIDs (diclofenac) (9). PD-1 inhibitors can also cause SJS (10), and the most reported agents were nivolumab and pembrolizumab. The most common drugs to cause chemotherapy-induced SJS include lenalidomide, methotrexate, docetaxel, and thalidomide. SJS happened concomitantly or within 8 weeks of the chemotherapeutic agents exposed. The immune checkpoint inhibitors latency period for inducing SJS/TEN ranged from 7 days to 140 days. A recent systematic review reported that SJS/TEN-like reactions caused by nivolumab had median onset time of 3 weeks in seven cases, whereas pembrolizumab had median onset time of 11 weeks in five cases, the average latency of SJS as 8.9 weeks (11). The patient had no drug allergies before, he had been treated with atorvastatin for several months, and he had no exposure to new compounds other than sintilimab. During the checkpoint inhibitor therapy, lichenoid drug eruption is one of common skin-related adverse reaction. Some cases are associated with severe erosive or bullous lesions. The lesions of bullous lichenoid drug eruption were epidermolytic, mild mucosal involvement, protracted disease course and relatively good overall health, but histological findings were not consistent with SJS. The histology of both lichenoid drug eruptions and SJS characterized by necrotic keratinocytes and subepidermal cleft formation. However, the more prominent lymphocytic infiltrate, along with jagged acanthosis (often with parakeratosis), is suggestive of lichenoid drug eruption. This histological findings in our case consistent with SJS (12, 13). Paraneoplastic syndrome can also manifest as cutaneous adverse reactions, but cutaneous manifestations usually occur months or years before tumor diagnosis (although they can appear simultaneously), and in this case the patient had no rash at the time of disease onset. Given the temporal relationship between initiation of immunotherapy and the onset of SJS, paraneoplastic SJS is less likely. Skin reactions caused by radiotherapy are usually localized radiation dermatitis in the radiation field. Radiotherapy may lead to a hypersensitivity reaction by preferentially impairing T suppressor cells. The hypersensitivity reaction induced by radiotherapy may have had synergistic and/or complementary contributions to the immune dysregulation. Many studies have shown that genetic factors contribute to differences in drug sensitivity. Chung et al. had demonstrated a strong association between the HLA-B*1502 and SJS induced by carbamazepine in Han Chinese (14). Furthermore, the HLA-A*02:07 and HLA-B*46:01 alleles were significantly associated with severe ocular complications among Han Chinese patients with SJS resulted by ICIs (15). However, genomic test for the patient did not detect these HLA subtypes. An MDT (multidisciplinary team) meeting was conducted by Dermatologists, pharmacologists, pathologists, oncologists and immunologists. Given the patient’s symptoms and history, SJS-induced by sintilimab was diagnosed, which is an uncommon and harmful adverse effect of sintilimab therapy. SJS is characterized by total epidermal necrosis due to extensive keratinocyte apoptosis.

Previously, sintilimab has been associated with myositis-myasthenia overlap syndrome (16), autoimmune diabetes mellitus (17), cytokine release syndrome, pulmonary fibrosis, hypothyroidism, and encephalitis (18–21). Now we report SJS caused by sintilimab. Knowing that the main cause of adverse reactions by ICIs is overactivation of immunity, but the mechanism is incompletely understood. Moreover, TMB, MSI, and dMMR were not present in the somatic cells; thus, we speculated whether it was the high expression of PD-L1 or PD-1 in the skin cells that led to SJS via the immune cells simultaneously attacking the tumor and the skin cells. Therefore, we performed tissue PD-1 and PD-L1 assays on the skin lesions, and the results showed that the skin biopsies were positive for both PD-1 and PD-L1 expression. PD-L1 is usually undetectable in skin cells, but anti-PD1 therapy could increase the expression of PD-L1 in keratinocytes and permit the activated CD8+ cytotoxic T cells to target keratinocytes, leading to keratinocyte apoptosis (22). Ziemer et al. also observed PD-L1 expression on the surface of deceased epidermal keratinocytes from all SJS/TEN patients (23). In the current patient, we detected PD-L1 expression in the glands and in the basal layer of the skin, and it appeared to be specific in the glands. Additionally, CD4 and CD8 positive T lymphocytes were infiltrated, indicating active immune response. CD8-positive T cells infiltration leaded to keratinocyte apoptosis and SJS. Whether more infiltration of immune cells after SJS is not clear and needs to be further studied in the future. Previous studies have suggested that the surge of PD-L1 expression in the epidermis may represent an antagonistic lymphocyte (22). The gene expression profile between anti-PD-1 medicines treated patients and SJS/TEN patients was similar, with upregulation of CXCL9, CXCL10, CXCL11, PRF1, GZMB, and FASLG. The relationship between dose or time of exposure to ICIs and cutaneous adverse reactions has not been fully elucidated (24). The onset of adverse reactions may continue for months or even years after discontinuation, and clinicians need to remain vigilant (25). The patient’s rash gradually improved through hormone shock therapy and infusion of immunoglobulin to achieve immunosuppressive and immunomodulatory effects, alleviating the patient’s symptoms.

In conclusion, sintilimab-associated SJS in lung cancer treatment is a very rare adverse event, but the consequences are serious. During and after treatment with anti-PD-1 agents, it is imperative to monitor the skin adverse reactions.

## Patient perspective

The patient expressed his gratitude to the medical staff for all their efforts during the treatment.

## Data availability statement

The datasets presented in this study can be found in online repositories. The names of the repository/repositories and accession number(s) can be found in the article/**Supplementary Material**.

## Ethics statement

Ethical review and approval was not required for the study on human participants in accordance with the local legislation and institutional requirements. The patients/participants provided their written informed consent to participate in this study. Written informed consent was obtained from the individual(s) for the publication of any potentially identifiable images or data included in this article.

## Author contributions

YZ and RW designed the concept and investigation. XL collected and analyzed data and wrote this paper, DC and GL analyzed data, LS provided assistance. All authors contributed to the article and approved the submitted version.
