# Supplementary material for: Case Report: sintilimab-induced Stevens-Johnson Syndrome in a patient with advanced lung adenocarcinoma
Source: Front Oncol. 2023 Sep 14;13:912168. doi: 10.3389/fonc.2023.912168 (PMC10540079; doi:10.3389/fonc.2023.912168)
Supplement: Supplementary file 1 [file Table_1.docx]

**Supplementary table S1. The topical and systemic agents employed for managing the skin adverse event**

| Agents | Time | Treatment method |
| --- | --- | --- |
| Fexofenadine Hydrochloride Tablets (120 mg oral daily) | From November 24, 2020 to November 25, 2020 | Systemic therapy |
| Compound glycyrrhizin Tablets (two tablets oral three times daily) | From November 24, 2020 to November 25, 2020 | Systemic therapy |
| Methylprednisolone (50mg intravenous infusion daily) | From November 26, 2020 to December 15, 2020 | Systemic therapy |
| Immunoglobulin (22.5g intravenous infusion daily) | From December 1, 2020 to December 4, 2020 | Systemic therapy |
| Prednisone (started at 40mg oral daily, then gradually reduced until discontinuation) | From December 16, 2020 to January 11, 2021 | Systemic therapy |
| Mupirocin ointment (moderate amounts application three times daily) | From November 26, 2020 to December 5, 2020 | Topical therapy |
| Halometasone Cream (moderate amounts application twice daily) | From November 26, 2020 to December 10, 2020 | Topical therapy |
| Fluticasone Propionate Cream (moderate amounts application daily) | From December 11, 2020 to January 5, 2021 | Topical therapy |
