# Supplementary material for: Case Report: sintilimab-induced Stevens-Johnson Syndrome in a patient with advanced lung adenocarcinoma
Source: Front Oncol. 2023 Sep 14;13:912168. doi: 10.3389/fonc.2023.912168 (PMC10540079; doi:10.3389/fonc.2023.912168)
Supplement: Supplementary file 2 [file DataSheet_1.docx]

**Supplementary Figure S1**


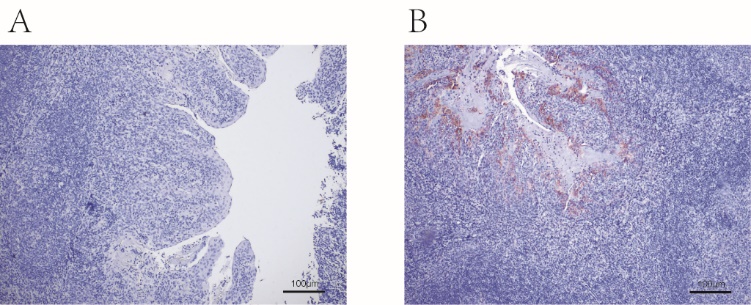


**Supplementary Figure 1** PD-L1 expression in tonsil tissue was used as a control to verify PD-L1 antibody specificity. (A) was negative control and (B) was positive control. The scale bar represents 100 μm.
